# Automatic extraction of reliable regions from multiple sequence alignments

**DOI:** 10.1186/1471-2105-8-S5-S9

**Published:** 2007-05-24

**Authors:** Timo Lassmann, Erik LL Sonnhammer

**Affiliations:** 1 Department of Cell and Molecular Biology, Karolinska Institutet, SE-171 77, Stockholm, Sweden; 2 Stockholm Bioinformatics Center, Stockholm University, S-106 91 Stockholm, Sweden

## Abstract

**Background:**

High quality multiple alignments are crucial in the transfer of annotation from one genome to another. Multiple alignment methods strive to achieve ever increasing levels of average accuracy on benchmark sets while the accuracy of individual alignments is often overlooked.

**Results:**

We have previously developed a method to automatically assess the accuracy and overall difficulty of multiple alignments. This was achieved by a per-residue comparison between alternate alignments of the same sequences. Here we present a key extension to this method, an algorithm to extract similarly aligned regions from several alignments and merge them into a new consensus alignment.

**Conclusion:**

We demonstrate that the fraction of correctly aligned residues within the resulting alignments is increased by 25 – 100 percent compared to the original input alignments, as only the most reliably aligned parts are considered.

## Background

Multiple alignments are of key importance in transferring annotation from model organism to humans [1]. The importance is reflected by the number of alignment methods that have emerged recently [2-5]. The development of alignment programs is governed by achieving ever increasing levels of accuracy on several commonly used benchmark sets [6,7]. The accuracy is usually measured by calculating the number of identically aligned residues divided by the number of aligned residues in a reference alignment. Essentially, this reflects the extent to which an alignment method managed to reconstruct a reference alignment. Misaligned residues in the test alignment are completely ignored. Therefore alignment programs that tend to align more residues, usually global methods, appear to perform well.

It is often more desirable in practice to create alignments in which only reliable regions are aligned and unreliable regions remain unaligned. Misaligned regions can give the impression of conservation where in fact there is none. This is particularly true in multi-domain cases where alignment programs often fail to insert large gaps corresponding to the loss, or gain, of entire domains. In phylogenetic reconstruction such misaligned regions essentially contribute noise that can lead to false tree topologies [8-10]. Previously, we made the simple observation that regions aligned similarly by several alignment methods are usually more reliable than those aligned differently [11]. Our method Mumsa takes advantage of this observation by using cross-alignment conservation as an indicator of alignment accuracy and overall case difficulty.

Here we present a new algorithm that extracts identically aligned regions from several multiple alignments and creates new alignments out of them. Within these alignments, unreliably aligned residues from the input alignments are disentangled and therefore we term them relaxed alignments.

## Algorithm

The input of our method is a set of multiple alignments of the same sequences generated either by different alignment methods or by a few methods employing different parameter settings.

An important concept in our algorithm are pairs-of-aligned residues (POARS) as defined in Lassmann and Sonnhammer (2005). Briefly, within an alignment two residues form a POAR when they occur in different sequences (rows) but within the same column.

The algorithm for the extraction of reliable regions from a set of alignments follows a few simple steps:

1. All input alignment in the set of alignments *M *are atomized into POARs.

2. The power set of *M*, *P*(*M*) is created. For example if |*M*| = 3, the following list of sub-sets is created: {},{A},{B},{C},{AB},{AC},{BC},{ABC} where A, B, C are alignments.

3. Each individual POAR is assigned to a single subset of *P*(*M*) depending on its occurrence within the input alignments. For example, an aligned pair of residues occurring in alignment A, B and C will be assigned to set {A, B, C} but not to any other set such as {A, B}.

4. Among the subsets of *P*(*M*) containing *f *alignments, select the one containing the maximum number of POARs (*X*). The parameter *f *is the stringency criterion for including residues in the final alignment: *f *= 3 requires all POARs in the final alignment occur in at least three input alignments, while *f *= 2 only requires POARs to occur in at least two alignments.

5. Build a set *Y *including all POARs that belong to *X *or sub-sets of *P*(*M*) of which *X *is a subset itself. If *X *= {A, B}, include POARs from this set but also from set {A, B, C}.

6. Assemble alignment by :

(a) Start with an alignment that contains only single residues in each column(i.e. an alignment where all sequences are completely unaligned)

(b) Sequentially merge columns if their residues form a POAR present in *y*.

(c) Sort the alignment columns to preserve the order of the residues in the initial sequences.

## Practical improvements

The previous version of Mumsa required that all input alignments to be in the Fasta format and contain the sequences in the same order. Both of these limitations were removed in this version to make it easier for users to use several different alignment methods.

Mumsa can produce output alignments in Fasta, Clustal and Macsim [12] alignment formats. When using the Macsim format, the residues in each sequence obtain a reliability score. For residue *A *this reliability score is the sum of the occurrences of all POARs, that *A *is involved in, normalized by the maximum reliability score attainable (the score if residue *A *would be aligned identically in all input alignments). These reliability scores can be visualized by Kalignvu [13] to aid users in distinguish between reliable and unreliable regions.

## Accuracy measurement

Alignment accuracy is measured by comparing a test alignment to a reference alignment. The sum-of-pairs score (SPS) [14] and Q-score [15] reflect how many residues are aligned identically in both test and reference alignment. In a sense this reflect how much of the reference alignment was reconstructed. However, no attention is being paid on the number of misaligned residues in the test alignment. For example a SPS score of 0.5 could either mean that 50 percent of the residues in the test alignment are simply not aligned but also that 50 percent are misaligned. We therefore introduce a per-residue accuracy (PRA) score that also has the number of shared POARs as the nominator but the total number of POARs of the test alignment as the denominator. Essentially, this is equivalent to asking how many residues are aligned correctly in the test alignment.

## Benchmark

To test our method we used a selection of alignment methods (Table 1) in combination with the Balibase benchmark set [6]. The strategy is as follows:

1. All 12 alignment programs are run on all test cases in Balibase.

2. Mumsa is run on the resulting alignments with varying stringency parameter *f*.

3. All alignments are scored using the PRA function.

In addition, we calculated the percentage of aligned residues, or the number of aligned residues divided by the total number of possible aligned residues.

The computational properties of Mumsa were tested by comparing the cumulative running time of alignment methods to the running time of Mumsa using three settings. We used the program ROSE [16] to generate sets of input sequences for the alignment methods. Since embarking on this project several alignment packages have been updated and we chose to adopted slightly more recent versions for this evaluation (Table 2). The CPU times were measured on a 2.0 GHz Xeon processor with 4 GB of RAM running Fedora Linux 6.0.

## Results and discussion

The PRA scores for all alignment methods used in this study are surprisingly low (Table 3). For reference set 11 all methods fail to align around half of the residues correctly while in other sets a full quarter of the residues are incorrectly aligned. However, in all cases the percentage of aligned residues for all alignment methods is high.

The relaxed alignments created by Mumsa have a much higher per-residue accuracy than those of the input alignment. Depending on the overall difficulty of the subset of benchmark alignments, the increase in accuracy is dramatic. This is especially true for the first Balibase reference set where the accuracy is almost doubled. It is particularly striking that the stringency cutoff *f *does not have to be high to give good accuracy gains. Residues occurring in more than 25 percent, here three or more, of input alignments are reliable and lead to good relaxed alignments.

As expected, the alignments generated by Mumsa contain fewer aligned residues than the input alignments. Moreover, the higher the cutoff *f *the fewer residues are aligned. Another observation is that the difficulty of the alignment cases affects the number of aligned residues in the merged alignments. For example, fewer aligned residues are present in the Mumsa alignments for the set 11, the most difficult one, compared to the other ones. In fact, the Pearson correlation coefficient between the average accuracy of input alignments (a measure of alignment difficulty) and the percentage of aligned residues of the most relaxed alignments is 0.98. This supports the notion that alignment programs usually disagree in difficult cases – more precisely in regions that are difficult to align.

**Figure 1:**
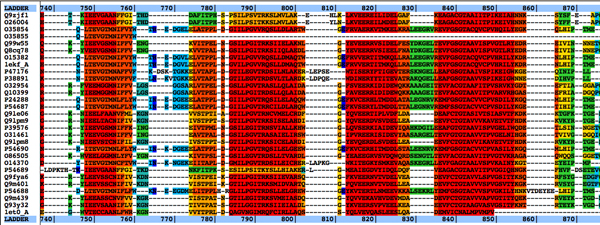
**A Mumsa alignment visualized by Kalignvu**. A relaxed Mumsa alignment derived from a ClustalW, Poa, Kalign, Probcons and Dialign alignment of the Balibase 3.0 test case BB20007. The parameter *f *was chosen to be two, requiring that residues in the output alignment appear in at least two input alignments. Each residue is colored according to the average occurrence of the POARs it is involved in. Regions that appear in red are identically aligned in all 5 input alignments while green and blue regions are only aligned identically in fewer and fewer cases. It is clear that all alignment programs find conserved motifs in the sequences but disagree on how the residues in between should be aligned.

The alignment viewer Kalignvu can be used to display the output alignments of Mumsa (Figure 1). A heat-map color scheme highlights the more reliable regions in red tones and less reliable regions in blue tones.

**Figure 2:**
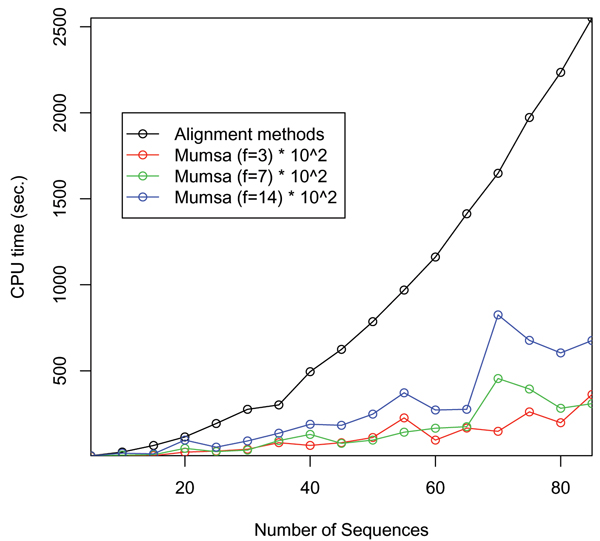
**Running time of Mumsa in comparison to the alignment methods used as input**. The running time in CPU seconds for Mumsa using three settings in comparison to the cumulative running time of the alignment programs used to generate the input alignments. The running times of Mumsa were multiplied by 100 to be visible in the plot. The sequence files were generated by ROSE [16] using an average sequence length of 500 residues and and average evolutionary distance of 250. It is clear that the running time of Mumsa is at least two orders of magnitude lower than that required by the alignment programs.

The running time of Mumsa is very low in comparison to that required by the input alignments (Figure 2). We ran Mumsa using a stringent, moderate and relaxed parameter setting for *f*. It is clear that the running time is influenced by the choice of *f*. Nevertheless, the running time of Mumsa remains two to three orders of magnitude lower than that required by the input alignment methods.

## Conclusion

Much emphasis has been given to alignment methods that focus on aligning a large fractions of residues because this often gives high scores on benchmark sets. We argue that it is equally relevant to increase the ration of correctly aligned residues within generated alignments. For this purpose we have introduced an algorithm to extract identically aligned parts from several multiple alignments and have shown that those are very reliable. Our method operates on pairs of aligned residues rather than columns. In addition to being able to identify blocks of high conservation, our method can therefore also identify correctly aligned regions which only few input sequences share.

In contrast to alignments generated by most popular direct alignment method, the relaxed alignments generated by Mumsa contain more gaps and are therefore less compact. As such they are inherently different from traditional alignments and users will have to decide whether these are suitable to be used directly in their specific studies. However, relaxed alignments can always be used to get an overview of conservation, a sense of how trustworthy alignments are and, more importantly, which regions are reliable. Users may wish to examine Mumsa alignments in a hierarchical manner, starting from the most reliable (high stringency cutoff *f*) to more compact but less reliable alignments (low *f*). Due to the good computationally properties of Mumsa little extra computing time is required to perform such an analysis. A direct application of relaxed alignments is in phylogenetics where alignment accuracy is of prime importance. An alignment in which 60 percent of the residues are aligned with an accuracy of more than 95 percent is clearly more desirable than an alignment where 90 percent of the residues are aligned, but incorrectly so in a quarter of cases.

## Availability and requirements

The Mumsa program is freely available at  or by request from T. Lassmann.

## Competing interests

The authors declare that they have no competing interests.

## Authors' contributions

TL had the idea of extracting similarly aligned regions from alternate alignments of the same sequences, implemented the method and carried out the evaluation. ELLS supervised the work. All authors read and approved the final manuscript.

## Figures and Tables

**Table 1:** Alignment methods and parameters used in this study.

Method	Description/Options
Poa [17,18]	local unprogressive mode using blosum80.mat
ClustalW version 1.83 [19]	default parameters
Muscle version 3.52 [15]	one iteration: -stable -maxiters 1
“	two iterations: -stable -maxiters 2
“	default: -stable
Probcons version 1.09 [2]	default parameters
Dialign version 2.2 [20,21]	default parameters
Mafft version 5.63 [3,22]	-localpair
“	-localpair -maxiterate 100
“	-globalpair
“	-globalpair -maxiterate 100
Kalign [4]	default parameters

**Table 2:** Alignment methods used to measure computational properties of Mumsa.

Method	Description/Options
Kalign	default parameters
ClustalW version 1.83	default parameters
“	-quicktree option
T-Coffee [23]	default parameters
Muscle version 3.6	default parameters
“	two iterations: -maxiters 1 -diags -sv -distance1
Probcons version 1.11	default parameters
Dialign version 2.2.1	default parameters
Mafft version 5.8613	-retree 1
“	-retree 2
“	-maxiterate 1000
“	-maxiterate 1000 -globalpair
“	-maxiterate 1000 -localpair
“	-maxiterate 1000 -genafpair

**Table 3:** PRA scores of various alignment methods for the Balibase subsets. Average PRA and percentage of aligned residues (in brackets) for several alignment methods on Balibase3. The average scores for all of the 12 alignment methods is given in the middle row (bold).

	Set 11	Set 12	Set 20	Set 30	Set 40	Set 50
Kalign	40.4 (91)	80.4 (94)	75.8 (94)	68 (92)	61.9 (89)	65.5 (89)
ClustalW	36.4 (97)	76.2 (96)	73.5 (95)	61.8 (94)	58.9 (92)	57.2 (93)
Muscle	41.8 (93)	80.6 (95)	76.3 (96)	68.5 (94)	62.6 (92)	64.4 (92)
Muscle_maxiters1	37.6 (91)	76.7 (95)	76.0 (95)	66.5 (93)	59.9 (91)	60.1 (91)
Muscle_maxiters2	39.2 (92)	78.5 (95)	76.0 (95)	67.7 (93)	61.8 (91)	62.9 (90)
G-INS-1	41.8 (87)	79.6 (94)	77.1 (94)	70.9 (91)	63.9 (88)	66.0 (89)
G-INS-i	44.7 (90)	81.1 (94)	78.1 (94)	71.8 (92)	64.8 (91)	68.1 (90)
L-INS-1	47.4 (89)	81.2 (94)	78.8 (93)	72.3 (92)	66.9 (89)	68.2 (89)
L-INS-i	50.3 (90)	82.7 (95)	79.8 (94)	73.1 (93)	68.1 (90)	69.7 (90)
Dialign	45.8 (72)	78.3 (91)	79.4 (88)	68.8 (85)	68.0 (79)	68.0 (81)
Probcons	49.8 (88)	83.3 (94)	79.8 (92)	72.9 (90)	69.0 (85)	71.6 (85)
Poa	46.3 (52)	78.5 (84)	75.3 (89)	62.9 (87)	63.6 (79)	58.2 (82)
Average	**43.5 (86)**	**79.7 (93)**	**77.2 (93)**	**68.8 (91)**	**64.1 (88)**	**65.0 (88)**
Mumsa (*f *= 1)	34.5 (93)	72.6 (93)	72.8 (94)	59.5 (91)	57 (90)	53.8 (90)
Mumsa (*f *= 2)	48.8 (73)	83.4 (85)	81.5 (86)	75.1 (81)	71.6 (76)	73.5 (77)
Mumsa (*f *= 3)	61.5 (49)	89.0 (78)	89.4 (77)	84.8 (68)	85.5 (60)	85.4 (60)
Mumsa (*f *= 4)	71.5 (39)	92.0 (74)	92.0 (72)	87.8 (64)	87.9 (58)	87.9 (59)
Mumsa (*f *= 5)	74.2 (34)	93.0 (73)	92.6 (72)	90.6 (62)	90.4 (54)	91.2 (55)
Mumsa (*f *= 6)	74.4 (29)	94.9 (67)	93.5 (70)	92.3 (58)	91.7 (53)	92.5 (52)
Mumsa (*f *= 7)	80.7 (27)	95.4 (66)	94.3 (69)	94.2 (55)	92.9 (50)	93.3 (50)
Mumsa (*f *= 8)	79.9 (24)	96.2 (63)	95.1 (67)	93.7 (55)	93.4 (49)	94.0 (49)
Mumsa (*f *= 9)	80.3 (23)	96.2 (63)	94.9 (67)	94.5 (55)	93.9 (48)	94.5 (47)
Mumsa (*f *= 10)	85.1 (21)	96.7 (62)	95.4 (66)	96.3 (50)	94.9 (46)	95.3 (45)
Mumsa (*f *= 11)	82.8 (18)	97.2 (59)	96.1 (63)	97.4 (47)	94.2 (44)	96.4 (42)
Mumsa (*f *= 12)	84.9 (13)	97.9 (53)	96.5 (60)	97.9 (43)	95.8 (39)	97.0 (37)
